# Prevalence of Punctal Stenosis Among Ophthalmology Patients

**DOI:** 10.4103/0974-9233.53867

**Published:** 2009

**Authors:** Amal Bukhari

**Affiliations:** From King Abdulaziz University Hospital, Jeddah, Saudi Arabia

**Keywords:** Blepharitis, Dry Eye, Epiphora, Prevalence, Punctal Stenosis

## Abstract

**Purpose:**

To estimate the prevalence of punctal stenosis among patients visiting the general ophthalmology clinic for routine checkup.

**Design:**

Prospective, observational case series.

**Materials and Methods:**

A total of 682 patients were evaluated for evidence of punctal stenosis from May to November 2008. Any associated findings from clinical examination were recorded.

**Results:**

As many as 54.3% (370/682) of the candidates had punctal stenosis. The prevalence is associated significantly with increasing age (p=.001), and no gender predilection was found. It was due to chronic blepharitis in 97% (359/370), entropion in 1.4% (5/370) and unknown causes in 1.6% (6/370) of the patients. As many as 58.1% (215/370) did not have subjective or objective evidence of epiphora, and all of them had a tear film breakup time of less than 10 seconds and positive corneal fluorescein staining.

**Conclusion:**

Punctal stenosis is a common finding among patients presenting for routine eye checkup. It increases with advancing age, and the most common predisposing factor is chronic blepharitis. A significant number of patients can be asymptomatic as they have concurrent dry eye disease. Surgical intervention is not recommended unless the patient is symptomatic after treating any associated blepharitis and dry eye disease.

## INTRODUCTION

Punctal stenosis can be congenital or acquired. The acquired type can result from a variety of causes, including lid trauma, malpositions, infections, tumors, blepharitis, toxic effects of some topical or systemic medications, autoimmune diseases like Stevens-Johnson syndrome and graft-versus-host reactions and from fibrosis secondary to punctal plug insertion.^1^–^7^ Aging was also found to be a predisposing factor for acquired punctal stenosis.^8^

The aim of this study was to estimate the prevalence of punctal stenosis among patients visiting the ophthalmology clinic for regular checkup and to determine the predisposing factors.

## MATERIALS AND METHODS

All patients that visited the general ophthalmology clinic from May to November 2008 were asked about any history of tearing, use of anti-glaucoma medications, systemic chemotherapy, facial radiotherapy, lacrimal surgeries and trauma. All underwent slit-lamp examination to evaluate the punctal size. Punctal stenosis was diagnosed using the criteria used by Kashkouli *et al.,*^9^,^10^ viz., a punctum that is visible but smaller than 0.3 mm and required a punctal finder followed by a standard punctal dilator to insert a #00 Bowman probe. Tear meniscus height was then measured using a slit-lamp biomicroscope. A level of more than 2 mm was considered high. Tear film breakup time (BUT) was also measured by touching the infero-temporal bulbar conjunctiva with a fluorescein sodium strip wetted with a preservative-free isotonic saline. Patients were asked to blink, and the precorneal tear film was examined under blue light illumination using a slit-lamp biomicroscope. The mean value of three measurements was recorded. The BUT was considered low when it was less than 10 seconds. The corneal surface was then examined for fluorescein staining and its presence or absence recorded. The cases were labeled asymptomatic if they did not complain of epiphora and their tear meniscus height was less than 2 mm. The patients were labeled as having dry eye if they had a BUT less than 10 seconds and positive corneal staining with fluorescein. A general ocular examination was then performed looking for associated diseases such as blepharitis, entropion, trichiasis, mass of the lacrimal drainage system and corneal or conjunctival scars.

The entire data was then analyzed using SPSS MS Window Release 11 (SPSS, Chicago, Illinois, USA). Using a Chi-square test, a ‘p’ value of less than 0.05 was considered significant

## RESULTS

Both eyes of the 682 candidates, with a mean age of 34.1 (range, 10-73 years), were evaluated for signs of punctal stenosis. Among them, 62.3% were females. As many as 370/682 (54.3%) patients had punctal stenosis. Table 1 shows the prevalence of punctal stenosis in relation to location, and Table 2 shows the prevalence in relation to age. As many as 51% (131/257) of the males and 56.2% (239/425) of the females were affected (p=0.18).

**Table 1:** Prevalence of Punctal Stenosis in Relation to Location

	Number of cases	%
Right upper punctual stenosis	231	33.9
Right lower punctal stenosis	151	22.1
Left upper punctal stenosis	245	35.9
Left lower punctal stenosis	146	21.4

**Table 2:** Prevalence of Punctal Stenosis in Relation to Age

Age group (Total number)	Number of cases	%
10-19 (130)	45	34.6
20-29 (231)	114	49.4
30-39 (70)	40	57.1
40-49 (121)	86	70.1
50-59 (60)	35	58.3
≥ 60 (70)	50	71.4
Total (682)	370	
*P* value	0.001	

Blepharitis was found in 359/370 (97%) of the cases (p=0.001). As many as 345/370 (93.2%) patients had dry eye disease (p=0.001), and 89.3% (308/345) of those dry eye patients had a form of chronic blepharitis (p=0.031).

As many as 155/370 (41.9%) patients gave history of tearing. All of them had a high tear meniscus height, 96.8% (150/155) of them had chronic blepharitis (p=0.042) and 20% (31/155) had dry eye disease (p=0.87). As many as 215/370 (58.1%) patients did not complain of tearing, and all had a normal tear meniscus height; while 88.8% (191/215) of the patients had chronic blepharitis (p=0.0281), and 97.2% (209/215) had dry eye (p=0.001).

An associated entropion was found in 5/370 (1.4%) patients, and 6/370 (1.6%) patients had no apparent cause for punctal stenosis. None of the patients had trichiasis, masses of the lacrimal drainage system, corneal or conjunctival scars. None of the patients were using anti-glaucoma medications, and none were on systemic chemotherapy No history of facial radiotherapy or lacrimal surgery or trauma was elicited.

## DISCUSSION

The prevalence of punctal stenosis was 54.3% among all the patients visiting the general eye clinic for routine checkup, which is considered a valid prevalence rate over the period of this study. It was found significantly correlated with increasing age, an observation that has also been noted by Kristan *et al.*^8^ and Kashkouli *et al.*^9^ This association is possibly due to aging changes and tissue atrophy, which can make the punctum a dense fibrous structure and less resilient. It can also make the surrounding orbicularis fibers atonic with a resultant punctal stenosis.^8^

In this study, gender was not found to have any statistically significant association with punctal stenosis, although Kashkouli *et al.*^9^ and Offutt *et al.*^14^ noted a female predominance among their patients (70% and 71%, respectively). They suggested that postmenopausal hormonal changes might account for this sex predilection.

Chronic blepharitis was the most common predisposing factor, affecting 97% of our patients. This observation is similar to that reported by Kashkouli *et al.,*^9^ who found that 45% of their patients had blepharitis. The authors suggested that chronic blepharitis might predispose to punctal stenosis, as as associated chronic inflammation would result in inflammatory membrane formation, conjunctival epithelial overgrowth and keratinization around the walls of the punctum. Edelstein *et al.*^11^ also correlated the recurrence of punctal stenosis after wedge punctoplasty to coexisting chronic blepharitis that causes causes punctal cicatrization.

As many as 58.1% of the cases lacked subjective or objective evidence of epiphora. Of all the cases, 88.8% had chronic blepharitis and 97.2% had dry eye disease. We believe that this group of patients did not have epiphora because of the increased tear evaporation from the associated dry eye disease which was caused by blepharitis. This observation made us suggest that having punctal stenosis might be an adaptation mechanism to compensate for the increased tear evaporation from the associated dry eye disease.

To conclude, punctal stenosis was a very common finding among our patients that were seen in the general eye clinic for routine checkup, a finding that would be expected to be higher if it was estimated among patients visiting the oculoplastic clinic. Punctal stenosis appeared to increase with advancing age, and the majority of patients had associated blepharitis. There were a significant number of asymptomatic patients and most of them had dry eye disease. We do not recommend surgical intervention for punctal stenosis in symptomatic patients until associated blepharitis or dry eye disease is well controlled.
